# IMF-Based MF and HS Energy Feature Information of F5, and F6 Movement and Motor Imagery EEG Signals in Delta Rhythms Using HHT

**DOI:** 10.3390/s23031078

**Published:** 2023-01-17

**Authors:** Chin-Feng Lin, Hong-Chang Lin

**Affiliations:** Department of Electrical Engineering, National Taiwan Ocean University, Keelung 20224, Taiwan

**Keywords:** HHT, IMF, MF, HS, MI, electroencephalogram, F5, F6

## Abstract

This study aims to extract the energy feature distributions in the form of marginal frequency (MF) and Hilbert spectrum (HS) in the intrinsic mode functions (IMF) domain for actual movement (AM)-based and motor imagery (MI)-based electroencephalogram (EEG) signals using the Hilbert–Huang transformation (HHT) time frequency (TF) analysis method. Accordingly, F5 and F6 EEG signal TF energy feature distributions in delta (0.5–4 Hz) rhythm are explored. We propose IMF-based and residue function (RF)-based MF and HS feature information extraction methods with *IMFRFERDD* (IMFRF energy refereed distribution density), *IMFRFMFERDD* (IMFRF MF energy refereed distribution density), and *IMFRFHSERDD* (IMFRF HS energy refereed distribution density) parameters using HHT with application to AM, MI EEG F5, and F6 signals in delta rhythm. The AM and MI tasks involve simultaneously opening fists and feet, as well as simultaneously closing fists and feet. Eight samples (32 in total) with a time duration of 1000 ms are extracted for analyzing F5AM, F5MI, F6AM, and F6MI EEG signals, which are decomposed into five IMFs and one RF. The maximum average *IMFRFERDD* values of IMF4 are 3.70, 3.43, 3.65, and 3.69 for F5AM, F5MI, F6 AM, and F6MI, respectively. The maximum average *IMFRFMFERDD* values of IMF4 in the delta rhythm are 21.50, 20.15, 21.02, and 17.30, for F5AM, F5MI, F6AM, and F6MI, respectively. Additionally, the maximum average *IMFRFHSERDD* values of IMF4 in delta rhythm are 39,21, 39.14, 36.29, and 33.06 with time intervals of 500–600, 800–900, 800–900, and 500–600 ms, for F5AM, F5MI, F6AM, and F6MI, respectively. The results of this study, advance our understanding of meaningful feature information of F5MM, F5MI, F6MM, and F6MI, enabling the design of MI-based brain-computer interface assistive devices for disabled persons.

## 1. Introduction

Extracting distinct feature information from electroencephalogram (EEG) signals becomes challenging when implementing brain computer interface (BCI) based systems of motor imagery (MI). These MI related BCI schemes can be applied to rehabilitation, assistive technology, and robotics. MI can be described as a “mental simulation or rehearsal of a movement without any motor output [1]”. Hortal [2] demonstrated that robotic mechanisms have been applied in telemanipulation, neuromotor control research, rehabilitation, and to assist impaired human motor control. These significant distinctive aspects of wearable robots facilitate intrinsic dual cognitive and physical interaction with humans. The IMF-based MF and HS time-frequency-energy feature information of F5, and F6 actual movement (AM) and MI EEG signals are significant research problems for BCI-based rehabilitation systems. In the explored process, the neural plasticity can be understood, an improvement at neurological level can be achieved, and the control commands of the BCI-based rehabilitation system can be generated. In addition, the activity of the brain can be reorganized by forming new neural connections. MI-based BCI systems aim to analyze the EEG measured without requiring direct AM of a human. MI-based BCI schemes can be used to send messages or commands to an electronic device only via brain activity instead of muscular activity; hence, they can serve as an alternative communication and control channel for humans with limited motor functions and improve their quality of life.

Yi et al. [3] investigated the differences in EEG signals with a three second time duration between simple and compound limb MI. They used event-related spectral perturbation, power spectral entropy and the spatial distribution coefficient to analyze the EEG signals. Their numerical results indicated distinct differences between simple and compound limb MI, which could be utilized to develop a MI based BCI system. Simple and compound limb tasks including both hands, left hand combined with the right foot, and right hand combined with the left foot movement were performed. MI can induce brain oscillations in the μ and beta rhythms, both of which originate in the sensorimotor cortex. Bethel et al. [4] studied the dynamics of sensorimotor activity during mental rotation induced via a hand laterality test (HLT). They investigated the differences between the left- and right-hand mental rotations by exploiting the time frequency technique. Their analysis results showed that the time frequency (TF) analysis of the EEG signal recorded during HLT is activities in the 8–12 and 12–24 Hz frequency bands. Kaneko et al. [5] studied whether the sensorimotor cortex of the brain during AM and MI activity depended on observed or imagined movement phases. A Fourier spectral analysis indicated that the alpha and beta power were reduced in the sensorimotor cortex during AM + MI and AM. Additionally, power spectral modulations were dependent on the walking phases.

Feature information extraction techniques have been developed in the time, frequency, and TF domains. Meziania et al. [6] proposed a lasso quantile periodogram based feature extraction method to recognize the spectrum of the MI related non-Gaussian, non-linear and non-stationary EEG signals. The proposed frequency analysis approaches employ discriminant information from these EEG signals of MI. The C3 and MI related EEG signal were analyzed. The EEG signal indicated activities in the 0–10 Hz frequency bands. Phothisonothai et al. [7] analyzed the EEG signal corresponding to the body part MI tasks in terms of feet, and index finger. The power spectrum distribution of C4 EEG data was analyzed using the fast Fourier transform method, and the analysis results indicate activity in 0–15 Hz bands. Abdalsalam et al. [8] demonstrated that BCI-based systems used to control devices have promoted new opportunity for disabled patients. The discrete wavelet transform feature extraction method was adopted to investigate F3, F4, T7, C3, C4, Cz, T8 and Pz EEG channel signals. Gonzalez-Rosaa et al. [9] extracted AM and MI EEG signals with task-related power parameters for sensorimotor (alpha and beta) rhythms. Fc1, Fc2, Fc5, Fc6, C3, C4, Cp1, Cp2, Cp5, Cp6, P3, P4, P7, and P8 EEG signals were applied while promoting motor learning experiment. The analysis results indicated the ability of MI to improve motor performance more than the physical practice alone.

The manner in which the features of MI-EEG are accurately extracted, has received increasing attention in the field of rehabilitation. Feature extraction methods are important because they directly affect the accuracy of MI-EEG signal task classification. An MI-EEG feature extraction method based on principal component analysis and deep belief networks has been proposed [10]. The method can achieve feature dimensionality reduction in EEG signals in a short time, has low algorithm complexity, and can extract higher signal features in MI-EEG. Akbar et al. [11] analyzed the potential features of MI-based C3, C4, and Cz EEG signals using low-pass filter banks with five different frequency banks between 0 and 5 Hz. Digital Butterworth low-pass filter banks with cut-off frequencies of 0.4, 1, and 2 Hz were illustrated, and the identification of neural activities from MI-EEG signals was achieved.

MI-EEG feature extraction aims to achieve the identification of motor intentions [12]. Consequently, the event related spectral perturbation maps of MI-based C3, C4, Cz, F1, and P6 EEG signals were obtained, and an interesting pattern of band event related synchronization was observed in 5–13, and 15–25 Hz bands. The MI practice has been applied as a behavioral strategy to prevent motor impairments owing to immobilization. Debarnot et al. [13] investigated the effects of MI training administered during 12 h arm immobilization on subsequent resting-state EEG power and coherence. The F3, F4, C3, C4, P3, and P4 MI-EEG signals were feature extracted, and the coherence power spectrum revealed coherence peaks in the delta (1–4 Hz) and alpha (8–12 Hz) bands. Guillermo et al. [14] proposed a kernel-based functional connectivity measure to address inter/intra-subject variability in MI and motor-related tasks. The functional connectivity between EEG channels were extracted with spatio-temporal-frequency patterns through their Gaussian kernel cross-spectral distribution. Singh et al. [15] described the current state of art techniques in data acquisition, MI training, preprocessing, feature extraction, channel and feature selection, and classification stages of the MI-BCI. The time, spectral, TF, spatial, spatio-temporal, and spatio-spectral domains, as well as Riemannian geometry feature extraction algorithms, were demonstrated.

Hilbert–Huang transformation (HHT) empirically based TF analysis method was proposed by Huang et al. [16,17], which could be applied to non-linear and non-stationary adaptive signal analysis processes, i.e., EEG. HHT comprises of empirical mode decomposition (EMD) and a Hilbert spectral analysis approach, and the basis functions are derived and adapted from the data employing EMD sifting procedures. Moreover, HHT analysis methods are data-dependent. Several intrinsic mode functions (IMFs) and one residual function (RF) are decomposed from the analysis data using EMD, where the number of IMFs is an HHT-based feature parameter. The instantaneous frequency (IF) is computed from derivatives of the phase functions of the Hilbert transform (HT) of the basis functions. HHT revealed the IMF and RF-based feature information in the TF space. Furthermore, HHT offers the advantage of high IMF and RF-based TF resolutions, while its disadvantages are high computational complexity and time requirement. Moreover, real-time application and design remains a challenge. The HHT’s power and effectiveness in data analysis have been demonstrated by its successful application to many important problems containing engineering, and biomedical data [18,19]. HHT has revealed the representation of the IMFs by B-spline functions, and filter-based decompositions have been generated. Additionally, they produce physically meaningful representations of biomedical signal from non-linear and non-stationary processes. In addition, the statistical significance characteristics information of the IMFs and one RF of biomedical signal have also been extracted. Guragai et al. [20] demonstrated several deep learning algorithms with good accuracy and stability to MI, these algorithms can easily process high-dimensional EEG signals. The power spectral density, continuous wavelet transform, fast Fourier transform, and short-time Fourier transform feature extraction methods were adopted in these learning algorithms. Lai et al. [21] presented the differences in wake and rapid eye movement sleep bruxism EEG signals with a duration of 60 s. The power spectral density analysis method was adopted, and a fast and high accuracy prognosis system for the sleep bruxism was developed. Heyat et al. [22] used the spectrum analysis method to detect S1 and rapid eye movement sleep bruxism EEG signals with a duration of 60 s. C4-P4 and C4-A1 EEG signals of healthy humans and bruxism patients were explored, and their accuracies were 81.70 and 74.11%, respectively.

Lin et al. [23] reviewed the application of HHT-based TF analysis to biomedical signals, such as EEG, electrocardiogram signals, electrogastrogram recordings, and speech signals, during the period from 1998 to 2011. The energy ratios of the clinical FP1, FP2, and Fz EEG waves of normal and alcoholic observers to its refereed total energy for IMF3, IMF4, IMF5, IMF6, IMF7, and the residual function have also been investigated [24]. The IFs of the clinical FP1, FP2, and Fz EEG waves of the normal and alcoholic observers were demonstrated. Additionally, the time-frequency-energy distributions of the IMF4 and IMF5 of the clinical FP1, FP2, and Fz EEG signals in the δ wave recorded from normal and alcoholic subjects observing two different pictures were revealed. The analysis time window of the clinical FP1, FP2, and Fz EEG signals was 20 s. The weighted average frequencies and magnitudes of the IMF1-IMF5 and RF for EEG signal without spike wave, and spike I, II, and III sample wave in epilepsy illness [25]. The analysis time window of spike sample waves was 1 s. Lin et al. [26] revealed an advanced HHT-based energy-frequency distributions corresponding to click signals of sperm whales. The spatial energy-frequency characteristics of IMFs, and one RF for the click I and II samples were explored. Subsequently, the energy characteristic distributions of the IMFs and RF for a blue whale sound signal with EMD were extracted [27]. High-resolution marginal frequency (MF) characteristics based on EMD with energy density intensity parameters for blue B call vocalizations, was presented.

Lee et al. [28] developed a novel feature extraction method that incorporated HHT and dispersion entropy to analyze MI-EEG signals. The features of IMFs 1 to 5 of C3 EEG signals with the time duration of 3 s in the 8–13 Hz frequency band were revealed. Yuyi et al. [29] proposed a feature extraction method of C3 and C4 EEG signals based on a subject executing left and right-hand MI using elliptical band-pass filter, HHT, and Hilbert entropy. The MF of μ and beta bands were isolated from the Hilbert spectrum (HS) of the selected IMFs of the filtered C3 and C4 EEG signals with time durations of 3 s. Trad et al. [30] extracted the features of C3 and C4 EEG signals with a time duration of 2 s by employing EMD and band power methods for MI-based BCI system design. In a previous physiological study [4], brain rhythms related to MI actions, known as sensorimotor rhythms, and primarily located in the mu and beta bands, were examined. Extensive analysis results showed that the characteristics of the active frequency bands corresponding to mu and beta band were only located in IMF1, and IMF2 on C3 and C4, respectively. Ortiz et al. [31] analyzed nine MI-based BCI EEG signal samples (Fc1, Fcz, Fc2, C1, Cz, C2, CP1, CPz, CP2) with time length of 100 s using EMD analysis method for lower-limb exoskeleton application. IMF1, IM2, IMF3, IMF4, and IMF5 were obtained in gamma, beta, alpha, theta, and delta rhythms.

The remainder of this paper is organized as follows: In Section 2, the HHT time-frequency analysis method is briefly described. In Section 3, the analysis results of the IMF-energy distribution, IMF-frequency energy distribution, and IMF-based TF energy distribution in the delta rhythms for F5MM, F5MI, F6MM, and F6MI BCI EEG signals are revealed in detail. Finally, the discussions and conclusions are presented in Section 4 and Section 5, respectively.

## 2. Materials and Methods

### 2.1. Datasets

F5 and F6 AM and MI EEG signals of S006 project with a time length of 120 s were downloaded from the EEG motor movement/imagery dataset [32] PhysioBank ATM (physionet.org, https://archive.physionet.org/cgi-bin/atm/ATM, accessed on 12 January 2023). It is an open and free EEG AM and MI dataset, and the EEG dataset were applied in previous studies [33,34]. S006 project recorded the EEG signals of healthy humans. The sample frequency was 160 Hz. AM considers that a target appears on either the top or the bottom of the screen. Subsequently, the subject opens and closes either both fists (if the target is on top) or both feet (if the target is on the bottom) until the target disappears.

Similarly, MI considers that a target appears on either the top or the bottom of the screen. Consequently, the subject imagines opening and closing either both fists (if the target is on top) or both feet (if the target is on the bottom) until the target disappears.

### 2.2. Methods

The proposed IMF-based MF and HS feature information extraction methods applied to MI EEG signals, are demonstrated as follows:

The EEG samples, denoted as *heeg*(*t*), are adaptively decomposed into *N* IMFs and one RF using the EMD method, as follows:

(1) $$heeg\left(t\right)=\sum_{i=1}^{N}IMF_{eegi}\left(t\right)+rf\left(t\right)$$

where $IMF_{eeg}\left(t\right)$ and *rf*(*t*) represent the *i*-th IMF and RF of the EEG samples, respectively.

The refereed total energy (RTE) of *heeg*(*t*) is given by

(2) $$E_{rte}=\sum_{i=1}^{N}IMF_{eegi}^{2}\left(t\right)+rf^{2}\left(t\right)$$

The energy ratio of the *i*-th IMF to the RTE of *heeg*(*t*), $IMFRE_{eegi}$ is defined as

(3) $$IMFRE_{eegi}=\frac{IMF_{eegi}^{2}\left(t\right)}{E_{rte}}\times 100\%$$

The energy ratio of the RF to the RTE of *heeg*(*t*), $RFRE_{eeg}$, is defined as

(4) $$RFRE_{eeg}=\frac{rf^{2}\left(t\right)}{E_{rte}}\times 100\%$$

The IMFRF energy refereed distribution density parameter, denoted as *IMFRFERDD,* is defined as follows:

(5) $$IMFRFERDD=\frac{100\%}{\left(N+1\right)}$$

Moreover, $z_{eegi}\left(t\right)$ is expressed as

(6) $$z_{eegi}\left(t\right)=IMF_{eegi}\left(t\right)+jHTIMF_{eegi}\left(t\right)$$

$$z_{eegi}\left(t\right)=A_{eegi}\left(t\right)e^{j\varphi_{eegi}\left(t\right)}$$

$$\;\;\;\;\;\;\;\;\;\;\;\;\;\;\;\;A_{eegi}\left(t\right)=\sqrt{IMF_{eegi}^{2}\left(t\right)+[HT{\left\{IMF_{eegi}\left(t\right)\right]}^{2}}$$

$$\varphi_{eegi}\left(t\right)={tan}^{-1}(\frac{HT\left\{IMF_{eegi}\left(t\right)\right\}}{IMF_{eegi}\left(t\right)})$$

$A_{eegi}\left(t\right)$, and $\varphi_{eegi}\left(t\right)$ represent the amplitude and the phase of $z_{eegi}\left(t\right)$, respectively. HT{ } indicates the HT.

The *i*-th *IF* of the EEG sample, *IFeegi*(*t*), is expressed as

(7) $$IF_{eegi}\left(t\right)=\frac{1}{2\pi }\frac{d\varphi_{eegi}\left(t\right)}{dt}$$

The MF of $IMF_{eegi}\left(t\right)$ in the m–n Hz band, that is, $MFRE_{eegimn}$ is calculated as

(8) $$MFRE_{eegimn}=\frac{IMF_{eegimn}^{2}\left(t\right)}{E_{rte}}\times 100\%$$

$IMF_{eegimn}^{2}\left(t\right)$ represents the energy of $IMF_{eegi}\left(t\right)$ in the m–n Hz band.

The *MF* of $RF_{eeg}\left(t\right)$ in the m–n Hz band, $MFRFRE_{eegmn}$*,* is calculated as

(9) $$MFRFRE_{eegmn}=\frac{rf_{eegmn}^{2}\left(t\right)}{E_{rte}}\times 100\%$$

$rf_{eegmn}^{2}\left(t\right)$ represents the energy of rf(t) in the m–n Hz band.

EEG signals include infra-low frequency (0–0.5 Hz), delta (0.5–4 Hz), theta (4–8 Hz), alpha (8–12 Hz), beta (12–30 Hz), and gamma (30–200 Hz) rhythms. The IMF and RF MF energy refereed distribution density parameter, denoted as *IMFRFMFERDD*, is defined as follows:

(10) $$IMFRFMFERDD=\frac{100\%}{\left(N+1\right)\times 6}$$

The HS of $IMF_{eegi}\left(t\right)$ in the m–n Hz band, during the time interval of $t_{1}$ and $t_{2}$, that is,

$HSRE_{eegimnt1t2}$ is calculated as

(11) $$HSRE_{eegimnt1t2}=\frac{IMF_{eegimnt1t2}^{2}\left(t\right)}{E_{rte}}\times 100\%$$

where $IMF_{eegimnt1t2}^{2}\left(t\right)$ represents the energy of $IMF_{eegi}\left(t\right)$ in the m–n kHz band, during the time interval of $t_{1}$ and $t_{2}$.

The HS of $RF_{eeg}\left(t\right)$ in the m–n Hz band, during the time interval of $t_{1}$ and $t_{2}$, $HSRFRE_{eegmnt1t2}$, is calculated as

(12) $$HSRFRE_{eegmnt1t2}=\frac{rf_{eegmnt1t2}^{2}\left(t\right)}{E_{rte}}\times 100\%$$

where $rf_{eegmnt1t2}^{2}\left(t\right)$ represents the energy of rf(t) in the m–n Hz band, during the time interval of $t_{1}$ and $t_{2}$.

EEG signals include *t* time intervals in the delta rhythm. The IMFRF HS energy refereed distribution density parameter, denoted as *IMFRFHSERDD*, is defined as follows:

(13) $$IMFRFHSERDD=\frac{100\%}{\left(N+1\right)\times 6\times t}$$

The *IMFRFERDD*, *IMFRFMFERDD*, and *IMFRFHSERDD* denote the IMFs and RF energy characteristic distribution, IMF and RF frequency-energy characteristic distribution, and IMF and RF time-frequency-energy characteristic distribution reference parameters, respectively. In the study, *N +* 1 and *t* equal 6 and 10, respectively. The *IMFRFERDD*, *IMFRFMFERDD*, and *IMFRFHSERDD* parameters in the study are 16.67, 2.78, and 0.278%, respectively. *IMFRFERDD* value of 2 denotes that the average ratios of the energy of IMF to RTE are 33.34%. *IMFRFMFERDD* value of 2 denotes that the average ratios of the energy of the IMF to the RTE in the delta rhythms are 5.56%. *IMFRFHSERDD* value of 2 denotes that the average ratios of energy of IMF to the RTE in the delta rhythm are 0.56% with a time length of 100 ms. The *IMFRFERDD*, *IMFRFMFERDD*, and *IMFRFHSERDD* parameters in the study are IMFs and RF-based energy distribution parameter, IMFs and RF-based frequency energy distribution parameter, and IMFs and RF-basedTF energy distribution parameter, respectively.

**Figure 1:**
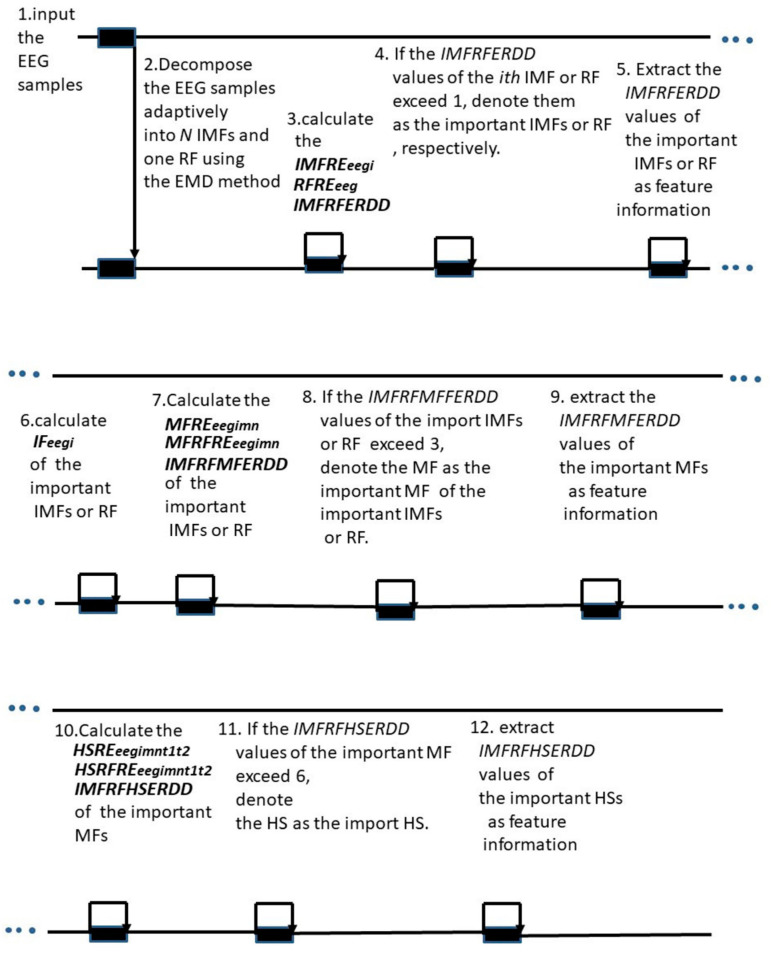
Schematic of the proposed feature extraction method.

The schematic of the proposed feature extraction method is shown in Figure 1. The proposed feature extraction method can be summarized as follows:

Step 1 Input the EEG samples.
Step 2 Decompose the EEG samples adaptively into *N* IMFs and one RF using the EMD method.
Step 3 Calculate the $IMFRE_{eegi}$, $RFRE_{eeg}$, and *IMFRFERDD* values.
Step 4 If the *IMFRFERDD* values of the *i*th IMF or RF exceed 1, denoted them as the important IMFs or RF, respectively.
Step 5 Extract the *IMFRFERDD* values of the important IMFs or RF as feature information.
Step 6 Calculate $IF_{eegi}$ of the important IMFs or RF.
Step 7 Calculate the $MFRE_{eegimn}$, $MFRFRE_{eegmn}$, and *IMFRFMFERDD* values of the important IMFs or RF.
Step 8 If the *IMFRFMFERDD* values exceed 3, denote the MF as the important MFs of the important IMFs or RF.
Step 9 Extract the *IMFRFMFERDD* values of the important MFs as feature information.
Step 10 Calculate the $HSRE_{eegimnt1t2}$, $HSRFRE_{eegmnt1t2}$, and *IMFRFHSERDD* values of the important MFs.
Step 11 If the *IMFRFHSERDD* values exceed 6, denote the HS as the important HS.
Step 12 Extract the *IMFRFHSERDD* values of the important HSs as feature information.

## 3. Results

**Figure 2:**
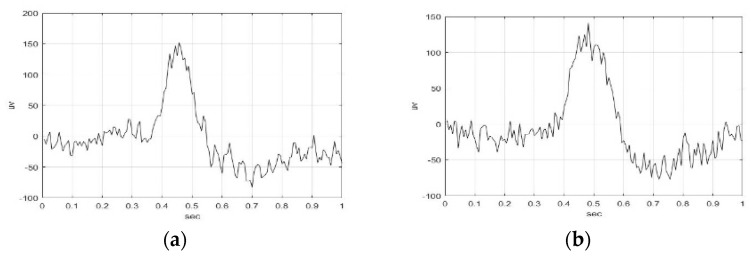

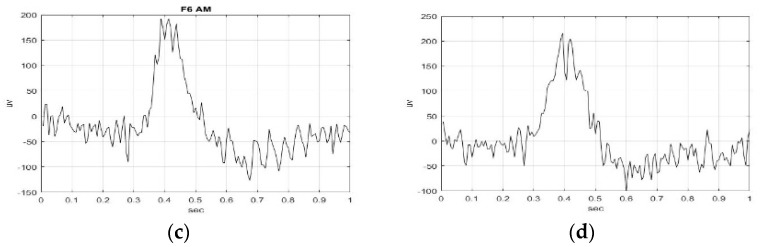
Sample 1(s) of F5AM, F5MI, F6AM, and F6MI EEG signal with time duration of 1 s. (**a**) F5AM, (**b**) F5MI, (**c**) F6AM, and (**d**) F6MI.

Eight samples each with time duration of 1 s were extracted for S006F5AM, S006F5MI, S006F6AM, and S006F6MI EEG signals, respectively. Sample 1 of S006F5AM, S006F5MI, S006F6AM, and S006F6MI EEG signals, respectively, are illustrated in Figure 2. The wave shape and structure of F5AM, F5MI, F6AM, and F6MI EEG sample 1 signals were obtained, and the analysis time window was 1 s. The maximum amplitudes of sample 1(s) of F5AM, F5MI, F6AM, and F6MI EEG signals are 152, 141, 192, and 172 μv, respectively, with F5 AM, and F6AM samples having larger values than those F5MI, and F6MI, respectively. 

Furthermore, the minimum amplitudes of the sample 1(s) of F5AM, F5R06, F6AM, and F6MI EEG signals are −83, −77, −127, and −98 μv, respectively, with F5AM, and F6AM samples having smaller values than those of F5MI and F6MI, respectively.

The Pearson’s correlation coefficient (PCC) (https://en.wikipedia.org/wiki/Pearson_correlation_coefficient, accessed on 12 January 2023) [35] was used to demonstrate the correlation of F5AM, F5MI, F6AM, and F6MI EEG signals, and is defined as:

(14) $$r=\frac{\sum_{i=1}^{N}(X_{i}-\bar{X)}(Y_{i}-\bar{Y)}}{\sqrt{\sum_{i=1}^{n}(X_{i}-{\bar{X)}}^{2}}\sqrt{\sum_{i=1}^{n}(Y_{i}-{\bar{Y)}}^{2}}}$$

*X*: F5AM, F5MI, F6AM, or F6MI EEG signal.

*Y*: F5AM, F5RMI, F6AM, or F6MI EEG signal.

The PCCs of the sample 1 of F5AM and F5MI, F5MI and F5MI, F5AM and F6AM, and F5MI and F6MI EEG signals, are 0.8231, 0.8479, 0.7545, and 0.5120, respectively. Additionally, the F5 AM and F5 MI, F5 MI and F5 MI, F5 AM and F6 AM, and F5 MI and F6 MI EEG signals were high (PCC value with 0.7 to 0.99) and middle (PCC value with 0.4 to 0.69) correlations, respectively.

### 3.1. Average IMF-Energy Distributions of F5AM, F5MI, F6AM and F6MI EEG Signals

**Figure 3:**
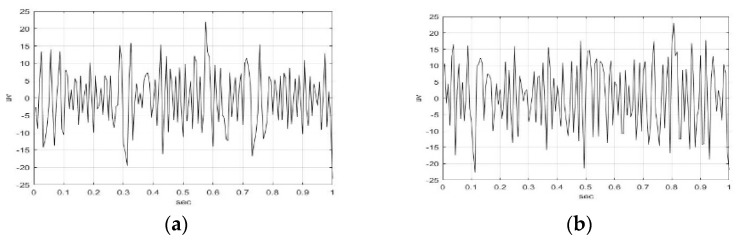

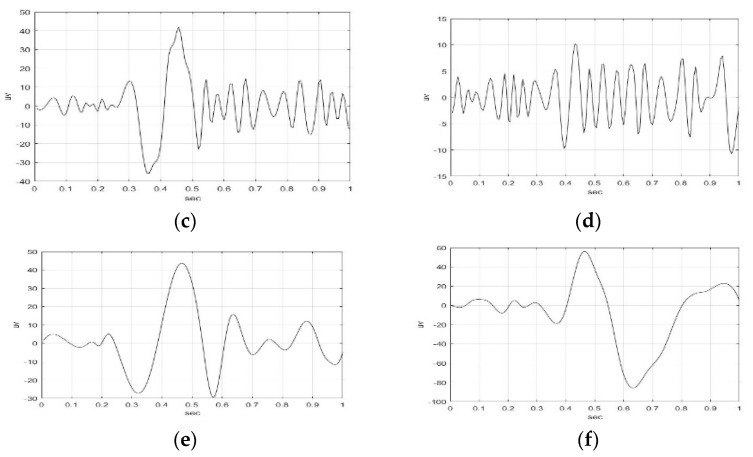
IMF1-IMF3 of F5AM and F5MI sample 1 EEG signals, respectively. (**a**) F5AM IMF1, (**b**) F5MI IMF1, (**c**) F5AM IMF2, (**d**) F5MI IMF2, (**e**) F5AM IMF3, and (**f**) F5MI IMF3.

**Figure 4:**
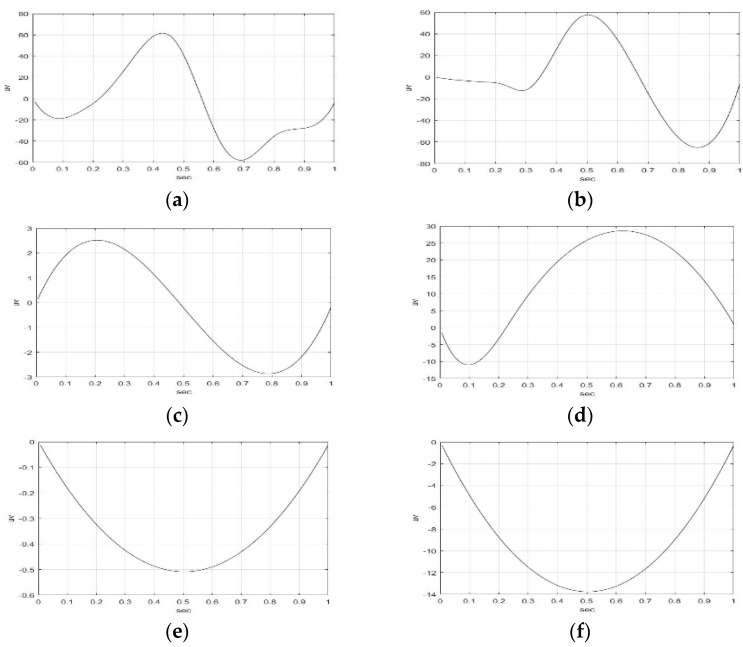
IMF4-IMF5, and RF of F5AM and F5MI sample 1 EEG signals, respectively. (**a**) F5AM IMF4, (**b**) F5MI IMF4, (**c**) F5AM IMF5, (**d**) F5MI IMF5, (**e**) F5AM RF, and (**f**) F5MI RF.

Figure 3 and Figure 4 illustrate the IMF1-IMF5, and RF of F5AM and F5MI sample 1 EEG signals using the EMD method. The wave shape, structure, and oscillation speed of IMF1-IMF5, RF of F5AM, and F5MI are obtained. F5AM and F5MI were decomposed into five IMFs and one RF using the EMD method. MATLAB-based EMD software was used in the research. The number of IMFs were automatic generated using MATLAB-based EMD software. The maximum amplitudes of IMF1-IMF5, and RF of F5AM were 21.97, 41.85, 43.65, 61.76, 2.52, and −0.01 μv, respectively. Meanwhile, the maximum amplitudes of IMF1-IMF5, and RF of F5MI were 23.11, 10.01, 56.32, 57.47, 28.59, and −0.34 μv, respectively, with IMF3 of F5MI, and IMF4 of F5AM samples having larger values than those of IMF3 of F5AM, and IMF4 of F5AM, respectively. 

Furthermore, the minimum amplitudes of IMF1-IMF5, and RF of F5AM are −23.20, −35.78, −29.65, −57.90, −2.87, and −0.51 μv, respectively, and those of IMF1-IMF5, and RF of F5MI are −22.64, −10.77, −85.81, −65.17, −10.94, and −13.76 μv, respectively. Notably, the minimum amplitudes of IMF3 and IMF4 of F5MI are smaller than those IMF3 and IMF4 of F5AM, respectively.

**Figure 5:**
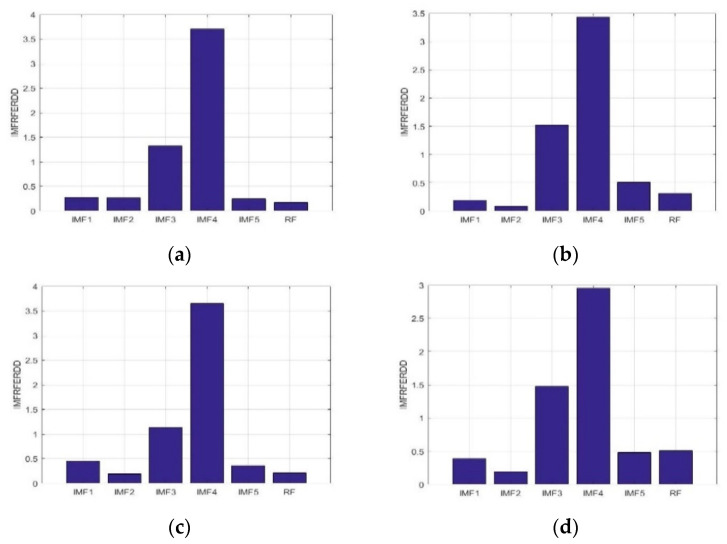
Average *IMFRFERDD* values of the IMF1-IMF5, and RF to the F5AM, F5MI, F6AM, and F6MI, respectively. (**a**) Average *IMFRFERDD* values (F5AM), (**b**) average *IMFRFERDD* values (F5MI), (**c**) average *IMFRFERDD* values (F6AM), and (**d**) average *IMFRFERDD* values (F6MI).

Eight samples each with time duration of 1 s were adaptively decomposed into IMF1-IMF5, and RF for S006F5AM, S006F5MI, S006F6AM, and S006F6MI EEG signals. Figure 5 presents the average *IMFRFERDD* values of IMF1-IMF5, and RF for F5AM, F5MI, F6AM, and F6MI, respectively. The average *IMFRFERDD* values of the IMF1-IMF5, and RF to the F5MM are 0.28, 0.27, 1.33, 3.70, 4.11, and 0.17, respectively; those of IMF1-IMF5, and RF are 0.19, 0.09, 1.52, 3.43, 0.50, and 0.31, respectively, for F5MI. Those of IMF1-IMF5, and RF are 0.46, 0.19, 1.13, 3.65, 0.36, and 0.21, respectively, for F6AM, and those of IMF1-IMF5, and RF are 0.39, 0.19, 1.48, 3.69, 0.48, and 0.51, respectively, for F6MI. Analysis results show that the important energy feature distributions are IMF3 and IMF4 for F5AM, F5MI, F6AM, and F6MI EEG signals. In addition, the maximum average *IMFRFERDD* values of IMF4 are 3.70, 3.43, 3.65, and 3.69 for F5AM, F5MI, F6AM, and F6MI, respectively. The second maximum average *IMFRFERDD* values of IMF3 are 1.33, 1.52, 1.13, and 1.48 for F5AM, F5MI, F6AM, and F6MI.

### 3.2. Average MF Feature Information of IMF3 and IMF4 for F5AM, F5MI, F6AM, and F6MI EEG Signals

**Figure 6:**
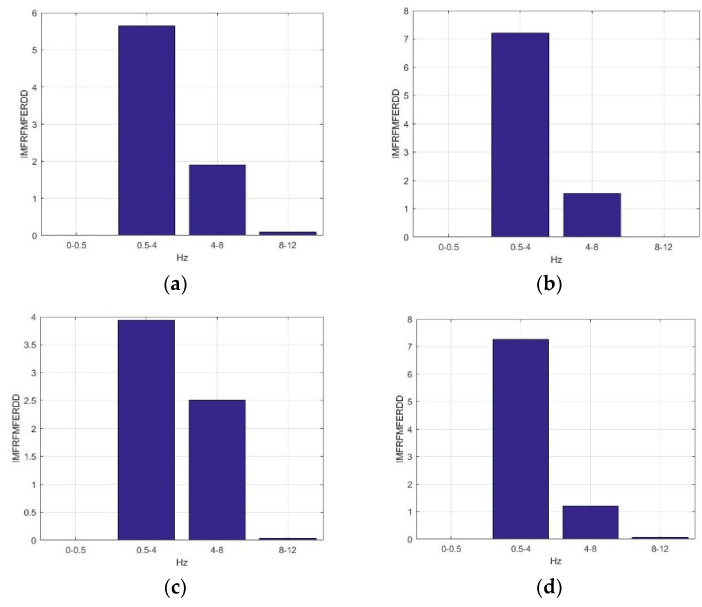
Average *IMFRFMFERDD* values of IMF3 (MF3) in ILF, delta, theta, and alpha rhythms for F5AM, F5MI, F6AM, and F6MI, respectively. (**a**) Average *IMFRFMFERDD* values (F5AM MF4), (**b**) average *IMFRFMFERDD* values (F5MI MF4), (**c**) average *IMFRFMFERDD* values (F6AM MF4), (**d**) average *IMFRFMFERDD* values (F6MI MF4).

Figure 6 presents the average *IMFRFMFERDD* values of IMF3 in ILF, delta, theta, and alpha rhythms for F5AM, F5MI, F6AM, and F6MI, respectively. Analysis results indicate that the critical energy feature distribution, IMF3, is in the delta and theta rhythms for F5AM, F5MI, F6AM, and F6MI EEG signals. The average *IMFRFMFERDD* values of IMF3 in delta rhythms are 5.64, 7.20, 3.94, 7,26, for F5AM, F5MI, F6AM, and F6MI, respectively, with *IMFRFMFERDD* values being larger than three for F5AM, F5MI, F6AM, and F6MI, respectively. Furthermore, the average *IMFRFMFERDD* values of IMF3 in theta rhythms are 1.90, 1.54, 2.50, 1.20, for F5AM, F5MI, F6AM, and F6MI, respectively, with *IMFRFMFERDD* values being larger than one, for F5AM, F5MI, F6AM, and F6MI, respectively. However, the average *IMFRFMFERDD* values of IMF3 in ILF and alpha rhythms are smaller than 0.1, for F5AM, F5MI, F6AM, and F6MI, respectively.

**Figure 7:**
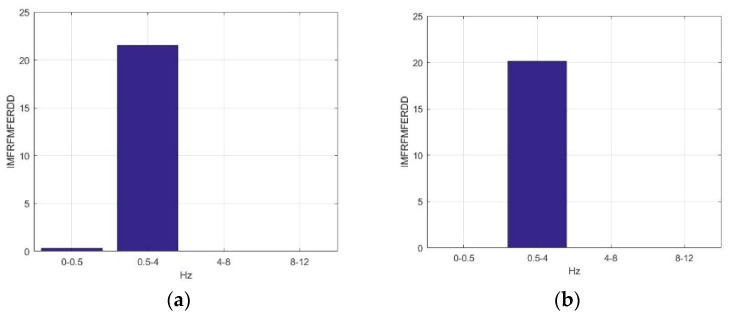

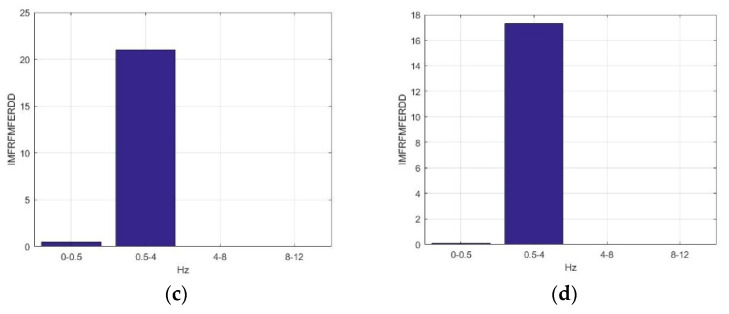
Average *IMFRFMFERDD* values of IMF4 (MF4) in ILF, delta, theta, and alpha rhythms for F5AM, F5MI, F6AM, and F6MI, respectively. (**a**) average *IMFRFMFERDD* values (F5AM MF4), (**b**) average *IMFRFMFERDD* values (F5MI MF4), (**c**) average *IMFRFMFERDD* values (F6AM MF4), and (**d**) average *IMFRFMFERDD* values (F6MI MF4).

Figure 7 presents the average *IMFRFMFERDD* values of IMF4 in ILF, delta, theta, and alpha rhythms for F5AM, F5MI, F6AM, and F6MI, respectively. Analysis results show that the critical energy feature distribution, IMF4, is in the delta rhythm for F5AM, F5MI, F6AM, and F6MI EEG signals, respectively. The average *IMFRFMFERDD* values of IMF4 in delta rhythms are 21.50, 20.15, 21,00, and 17.30, for F5AM, F5MI, F6AM, and F6MI, respectively, with *IMFRFMFERDD* values being larger than 17 for F5AM, F5MI, F6AM, and F6MI, respectively. In contrast, the average *IMFRFMFERDD* values of IMF4 in the ILF rhythms are smaller than 0.54, for F5AM, F5MI, F6AM, and F6MI, respectively. Moreover, the average *IMFRFMFERDD* values of IMF4 in the theta, and alpha rhythms are smaller than 0.02, for F5AM, F5MI, F6AM, and F6MI, respectively.

### 3.3. Average HS Feature Information of IMF3 and IMF4 for F5AM, F5MI, F6AM, and F6MI EEG Signals

**Figure 8:**
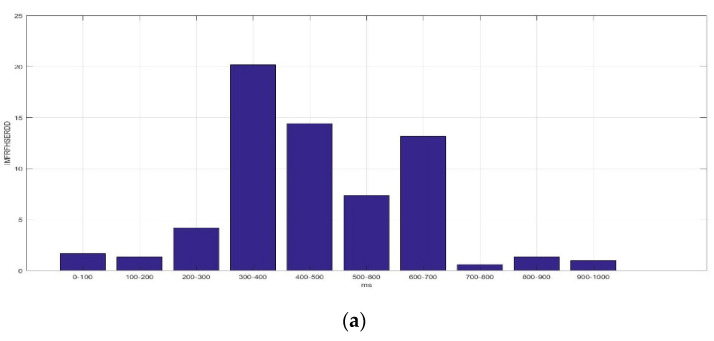

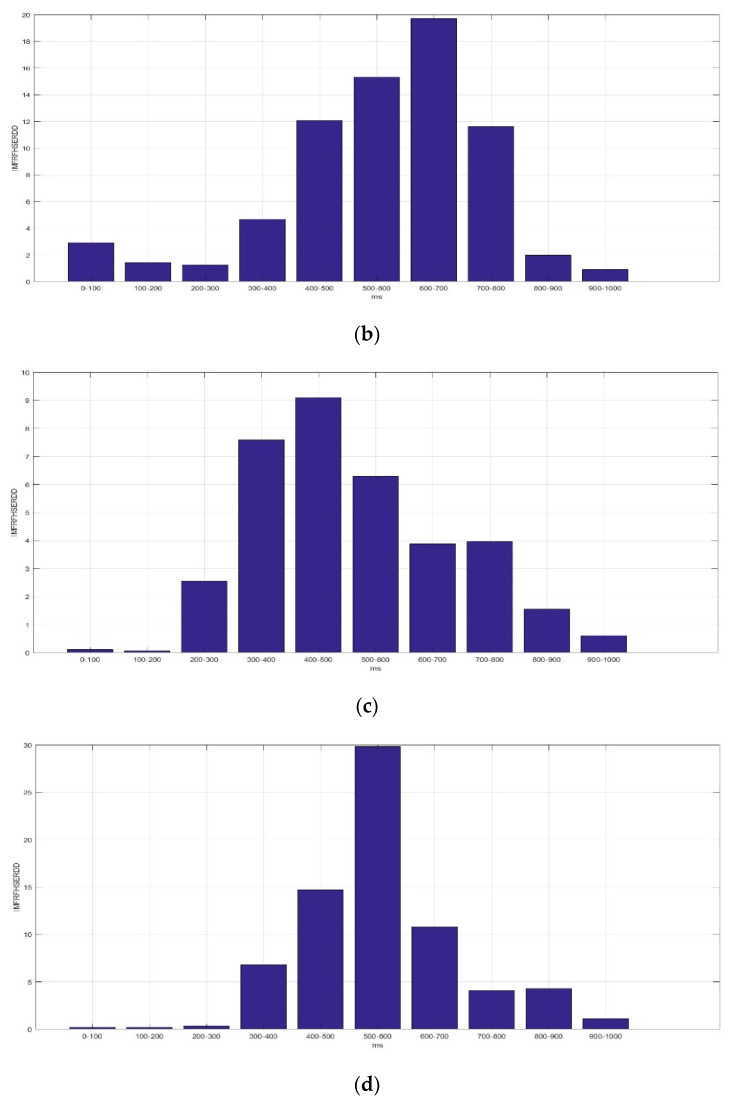
Average *IMFRFHSERDD* values of IMF3 (HS3) for the delta rhythm during time interval of 0–1000 ms for F5AM, F5MI, F6AM, and F6MI, respectively. (**a**) Average *IMFRFHSERDD* values (F5AM HS3, 0.5–4 Hz), (**b**) average *IMFRFHSERDD* values (F5MI HS3, 0.5–4 Hz), (**c**) average *IMFRFHSERDD* values (F6AM HS3, 0.5–4 Hz), and (**d**) average *IMFRFHSERDD* values (F6MI HS3, 0.5–4 Hz).

Figure 8 presents the average *IMFRFHSERDD* values of IMF3 (HS3) in the delta rhythm during the time interval of 0–1000 ms for F5AM, F5MI, F6AM, and F6MI, respectively. Analysis results show that the critical energy feature distribution, IMF3, is in the delta rhythm during the time interval 300–600 ms for F5AM and F6AM, EEG signals. The critical energy feature distribution, IMF3, is in the delta rhythm during the time interval 400–700 ms for F5MI and F6MI, EEG signals. The average *IMFRFHSERDD* values of IMF3 in the delta rhythm during the time intervals 200–300, 300–400, 400–500, 500–600, and 600–700 ms are 4.20, 20.18, 14,42, 7.37, and 13.17 for F5AM, respectively. Furthermore, the average *IMFRFHSERDD* values of IMF3 in the delta rhythm during time intervals 300–400, 400–500, 500–600, 600–700, and 700–800 ms are 4.68, 12.09, 15.32, 19.71, and 11.62 for F5MI, respectively. In addition, the average *IMFRFHSERDD* values of IMF3 in the delta rhythm during the time intervals 300–400, 400–500, 500–600, 600–700, and 700–800 ms are 7.59, 9.10, 6.29, 3.88, and 3.96 for F6AM, respectively. Finally, the average *IMFRFHSERDD* values of IMF3 in the delta rhythm during the time intervals 300–400, 400–500, 500–600, 600–700, and 700–800 ms are 6.80, 14.71, 29.82, 10.83, and 4.10 for F6MI, respectively. 

The average *IMFRFHSERDD* values of IMF3 in delta rhythm are larger than seven during the time intervals 300–400, 400–500, 500–600, and 600–700 ms, for F5AM, respectively. Meanwhile, those of IMF3 are larger than seven during the time intervals 400–500, 500–600, 600–700, and 700–800 ms for F5MI, respectively. Furthermore, those of IMF3 are larger than seven during the time intervals 300–400 and 400–500 ms, for F6AM, respectively. Those of IMF3 are larger than 7 during the time intervals 400–500, 500–600, and 600–700 ms, for F6MI, respectively. Additionally, those of IMF3 are larger than seven during the time intervals 400–500 and 500–600 ms, for F6MI, respectively, and those of IMF3 are larger than seven during the time intervals 300–500 ms, for F5MM and F6MM, respectively. Finally, those of energy of IMF3 to the RTE are larger than seven during the time intervals 400–700 ms, for F5MI and F6MI, respectively.

**Figure 9:**
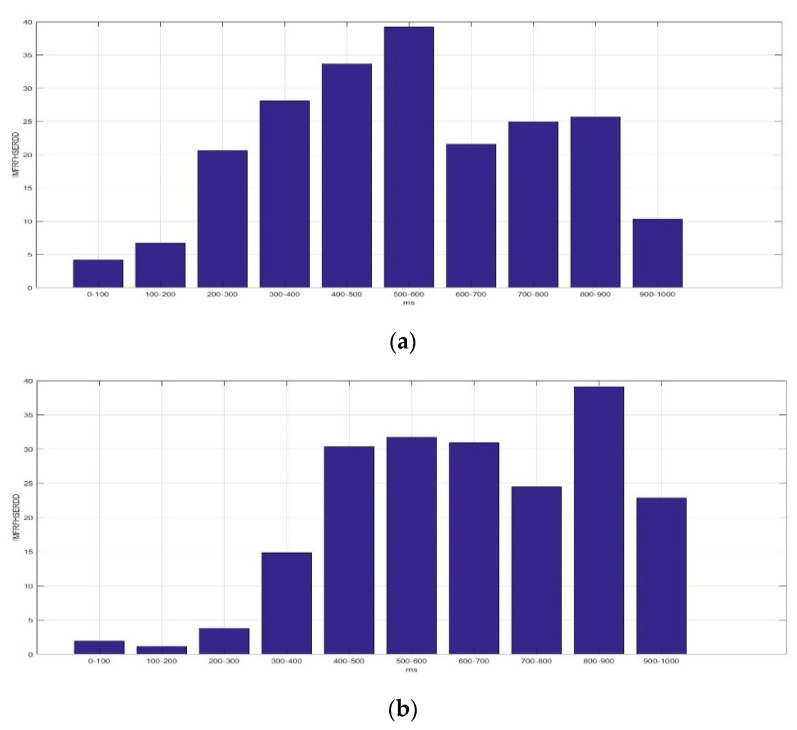

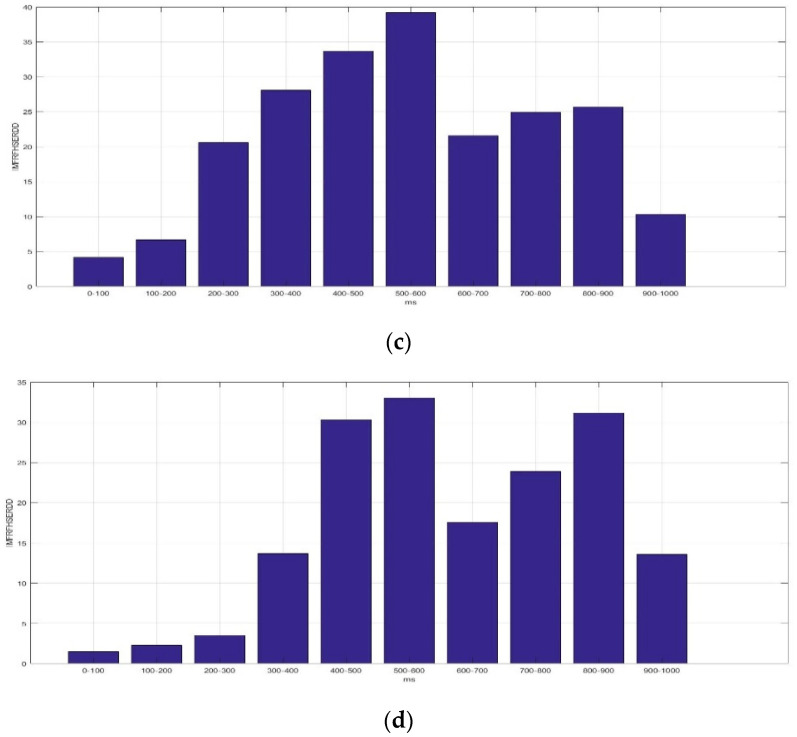
Average IMFRF HS energy refereed distribution density of IMF4 (HS4) for the delta rhythm during time interval of 0–1000 ms for F5AM, F5MI, F6AM, and F6MI, respectively. (**a**) Average *IMFRFHSERDD* values (F5AM HS4, 0.5–4 Hz), (**b**) average *IMFRFHSERDD* values (F5MI HS4, 0.5–4 Hz), (**c**) average *IMFRFHSERDD* values (F6AM HS4, 0.5–4 Hz), and (**d**) average *IMFRFHSERDD* values (F6MI, HS4 0.5–4 Hz).

Figure 9 presents the average *IMFRFHSERDD* values of IMF4 (HS4) in the delta rhythm during the time interval 0–1000 ms for F5AM, F5MI, F6AM, and F6MI, respectively. Analysis results show that the critical energy feature distribution, IMF4, is in the delta rhythm during the time interval 200–1000 ms for F5AM and F6AM EEG signals. The critical energy feature distribution, IMF4, is in the delta rhythm during the time interval 300–900 ms for F5MI and F6MI EEG signals. The average *IMFRFHSERDD* values of the IMF4 in the delta rhythm during the time intervals 200–300, 300–400, 400–500, 500–600, 600–700, 700–800, 800–900, and 900–1000 ms are 20.61, 28.09, 33.67, 39.21, 24.93, 25.68, and 10.32 for F5AM, respectively. Furthermore, those of IMF4 during the time intervals 300–400, 400–500, 500–600, 600–700, 700–800, 800–900, and 900–1000 ms are 14.82, 30.36, 31.73, 30.90, 24.50, 39.14, and 22.84 for F5MI, respectively. In addition, those of IMF4 during the time intervals 200–300, 300–400, 400–500, 500–600, 600–700, 700–800, 800–900, and 900–1000 ms are 7.99, 13.67, 31.69, 35.58, 22.95, 30.83, 36.29, and 18.42 for F6AM. Furthermore, those of IMF4 during the time intervals 300–400, 400–500, 500–600, 600–700, 700–800, 800–900, and 900–1000 ms are 13.67, 30.32, 33.05, 17.59, 23.88, 31.18, and 13.56 for F6MI. Thus, the average *IMFRFHSERDD* values of IMF4 in the delta rhythm during the time intervals 300–900 ms are larger than 18 for F5AM. Meanwhile, those of IMF4 during the time interval of 400–1000 ms are larger than 18 for F5MI. In addition, those of IMF4 during the time interval 400–1000 ms are larger than 18 for F6AM, and those of IMF4 in the delta rhythm during time intervals of 400–600 and 700–900 ms are larger than 18 for F6MI. 

## 4. Discussions

Table 1 lists the comparison of features extracted methods following application to MI-based EEG signals. Feature information of AM and MI EEG signals is an interesting research topic. In previous studies, the feature information of C3, C4, and Cz MI EEG signals with the alpha and beta rhythms has been discussed. The analysis time lengths of MI EEG signals are larger than 1 s. In this study, the feature information of F5, and F6 MI EEG signals with the delta rhythms was explored, by adopting the HHT-based analysis method. The frontal lobe area of the brain that controls movement is responsible for controlling finger, foot, and tongue movements. The analysis time length is 1000 ms. With an increase in the analysis time length of EEG signals, the computational complexity of the EMD process increased, and consequently, real-time application becomes challenging. The analysis time length of 1000 ms is a good parameter for real-time analysis. Previous studies have rarely explored, F5AM, F5MI, F6AM, and F6MI EEG signals with a time duration of 1000 ms in delta rhythms. In this study, eight samples each with time duration of 1000 ms were extracted and analyzed for S006F5AM, S006F5MI, S006F6AM, and S006F6MI EEG signals, respectively.

The samples were visually approximated for the samples of F5AM, F5MI, F6AM, and F6MI EEG signals. The F5AM and F5MI, F6AM and F6MI, F5AM and F6AM, and F5MI and F6MI EEG samples were high, high, high, and middle PCC correlations, respectively.

Eight samples each with time duration of 1000 ms, were adaptively decomposed into five IMFs and one RF for F5AM, F5MI, F6AM, and F6MI EEG signals, respectively. Table 2 lists the IMF-based MF and HS energy feature information for F5AM, F5MI, F6AM, and F6MI EEG signals. The maximum average *IMFRFERDD* values of F5MI and F6MI IMF4 are similar to those of the F5AM and F6AM IMF4, respectively. However, the second maximum average *IMFRFERDD* values of the F5MI and F6MI IMF3 are larger than those of the F5AM and F6AM IMF3, respectively.

The MFs of IMF3 exhibit the maximum average *IMFRFMFERDD* values in the delta rhythms for F5AM, F5MI, F6AM, and F6MI, respectively. Additionally, the MFs of IMF3 exhibit the second maximum average *IMFRFMFERDD* values in the theta rhythms for F5AM, F5MI, F6AM, and F6MI, respectively. Moreover, the MFs of IMF4 (MF4) exhibit the maximum average *IMFRFMFERDD* values in the delta rhythms for F5AM, F5MI, F6AM, and F6MI, respectively. However, the maximum average *IMFRFMFERDD* values of MF4 of F5AM, and F6AM are larger than those of MF4 of F5MI, and F6MI in the delta rhythm, respectively.

The maximum average *IMFRFHSERDD* values of IMF3 in the delta rhythm are 20.18, 19.71, 9.10, and 29,82 with time intervals 300–400, 600–700, 400–500, and 500–600 ms, for F5AM, F5MI, F6AM, and F6MI, respectively. Furthermore, the second maximum *IMFRFHSERDD* values of IMF3 in the delta rhythm are 14,42, 15.32, 7.59, and 14.71 with time intervals 400–500, 500–600, 300–400, and 400–500 ms, for F5AM, F5MI, F6AM, and F6MI, respectively.

The maximum average *IMFRFHSERDD* values of IMF4 in the delta rhythm are 39.21, 39.14, 36.29, and 33.06 with time intervals 500–600, 800–900, 800–900, and 500–600 ms, for F5AM, F5MI, F6AM, and F6MI, respectively. In addition, the second maximum average *IMFRFHSERDD* values of IMF4 in the delta rhythm are 33.67, 31.73, 35.58, and 31.19 with time intervals 400–500, 500–600, 500–600, and 800–900 ms, for F5AM, F5MI, F6AM, and F6MI, respectively. Finally, the third maximum average *IMFRFHSERDD* values of IMF4 in the delta rhythm are 28.09, 30.90, 31.69, and 30.90 with time intervals 300–400, 600–700, 700–800, and 400–500 ms, for F5AM, F5MI, F6AM, and F6MI, respectively. Thus, these analysis results reveal the *IMFRFERDD* values distributions, *IMFRFMFERDD* values of IMF3, *IMFRFMFERDD* values of IMF4, *IMFRFHSERDD* values of IMF3, and *IMFRFHSERDD* values of IMF4 in the delta rhythm features information.

## 5. Conclusions

MI-based control is highly correlated with the F5 and F6 position neurons activities of the brain. This study was conducted to extract the time-frequency-energy feature information distributions of F5AM, F5MI, F6AM, and F6MI, using the HHT TF analysis method. The *IMFRFERDD* values of the IMF distribution ratios, *IMFRFMFERDD* values of IMFs, and *IMFRFHSERDD* values of IMFs in the delta rhythms of F5AM, F5MI, F6AM, and F6MI, were extracted. The maximum average *IMFRFMFERDD* values of IMF3 in the delta rhythms are 5.64, 7.20, 3.94, and 7.26 for F5AM, F5MI, F6AM, and F6MI, respectively. The maximum average *IMFRFMFERDD* values of IMF4 in the delta rhythms are 21.50, 20.15, 21, and 17.30 for F5AM, F5MI, F6AM, and F6MI, respectively. The maximum average *IMFRFMFERDD* values of IMF4 for F5AM; those for F6AM are larger than those for F5MI, and F6MI in the delta rhythm. Thus, the results of this study can facilitate better understanding of the meaningful feature information of MI-based BCI design, and can be applied to MI-based BCI bimanual prosthesis and prosthetic feet control. More specifically, the IMF-derived MI features can be applied to detection, classification, and identification BCI-based EEG neuromotor control system, to assist and increase impaired human motor control accuracy.

## Figures and Tables

**Table 1:** Comparison of features extracted methods with application to MI-based EEG signals.

	EEG Channels	Analysis Time Window	Analysis Method	Characteristics Rhythm	MI Task
Yi et al. [2]	C3, Cz, C4	10 s	Short time Fourier transform Event-related spectral perturbation Power spectral entropy	μ rhythm (7.5–12.5 Hz) Central beta rhythm (14–29 Hz)	Three tasks of compound limb motor imagery (both hands, left hand combined with right foot, right hand combined with left foot) and rest state.
Bethel et al. [3]	C3, C4	2.2 s	Short time Fourier transform Time-frequency power spectral	3–40 Hz	Three tasks of right counter-clockwise by 90 degree (CCW) hand laterality test (HLT), left clock wise by 90 degree (CW) HLT, right CW HLT and left CCW HLT.
Meziania et al. [5]	C3	2 s	FFT	Mu rhythm (8–12 Hz) Beta rhythm (18–25 Hz).	Left hand movement
Cheng et al. [9]	C3, Cz, C4	9 s	Principal component analysis (PCA) Deep belief networks (DBN)	0.5–30 Hz	Left and right hands
Akbar et al. [10]	C3, Cz, C4	1200 s	Low-pass filter banks	0–5 Hz	Hand movement
Andrade et al. [11]	C3, C4, Cz, F1, P6	4 s	Short time Fourier transform event related Spectral perturbation maps Time-frequency power spectral	1.5–13 Hz 2.15–25 Hz	Train disorders of action and intention understanding beyond simple imitation, such as autism.
Debarnot et al. [12]	F3, F4, C3, C4, P3, P4	12 h	The coherence power spectrum revealed coherence peaks	delta (1–4 Hz) band. alpha (8–12 Hz) bands	Training administered during 12 h arm immobilization on subsequent resting-state
Lee et al. [22]	C3	3 s	EMD IMF	8–13 Hz band	Data driven information theoretic feature extraction
Yuyi et al. [23]	C3, C4	3 s	HHT Hilbert entropy.	beta rhythm (18–25 Hz).	Executing left and right-hand
Trad et al. [24]	C3, C4	2 s	EMD Band power methods	mu rhythm (8–12 Hz) beta rhythm (18–25 Hz)	BCI system design
Ortiz et al. [25]	Fc1, Fcz, Fc2, C1, Cz, C2, CP1, CPz, CP2	100 s	EMD IMF	gamma, beta, alpha, theta, and delta rhythms.	Lower-limb exoskeleton application
This study	F5, F6	1 s	EMD IMF IMFRFERDD, IMFRFMFERDD, IMFRFHSERDD	delta, theta rhythms.	Open fists and feet at the same time, as well as close fists and feet at the same time.

**Table 2:** IMF-based MF and HS energy feature information for the F5AM, F5MI, F6AM, and F6MI EEG signals.

	F5AM	F5MI	F6AM	F6MI
main IMFs (*IMFRFERDD* values)	IMF3 (3.70) IMF4 (4.11)	IMF3 (1.52) IMF4 (3.43)	IMF3 (1.13) IMF4 (3.65)	IMF3 (1.48) IMF4 (3.69)
main MF3 (*IMFRFMFERDD* values)	delta rhythms (5.64)	delta rhythms (7.20)	delta rhythms (3.94)	delta rhythms (7.26)
main MF3 (*IMFRFMFERDD* values)	theta rhythms (1.90)	theta rhythms (1.54)	theta rhythms (2.50)	theta rhythms (1.20)
main MF4 (*IMFRFMFERDD* values)	delta rhythms (21,50)	delta rhythms (20.15)	delta rhythms (21.00)	delta rhythms (17.30)
main HS3 delta rhythms (*IMFRFHSERDD* values)	300–400 ms (20.18) 400–500 ms (14.41) 600–700 ms (13.17)	400–500 ms (12.09) 500–600 ms (15.32) 600–700 ms (19.71) 700–800 ms (11.62)	300–400 ms (7.59) 400–500 ms (9.10) 500–600 ms (6.29)	400–500 ms (14.71) 500–600 ms (29.82) 600–700 ms (10.83)
main HS4 delta rhythms (*IMFRFHSERDD* values)	200–400 ms (48.70) 400–600 ms (72.88) 600–800 ms (46.43) 800–1000 ms (36.00)	300–500 ms (45.18) 500–700 ms (62.63) 700–900 ms (63.64)	200–400 ms (21.66) 400–600 ms (67.27) 600–800 ms (53.78) 800–1000 ms (54.71)	300–500 ms (43.99) 500–700 ms (50.64) 700–900 ms (55.06)

## Data Availability

Not applicable.

## Abbreviations

AM	actual movement
BCI	brain computer interface
EEG	electroencephalogram
EMD	empirical mode decomposition
HLT	Hand laterality test
HT	Hilbert transform
HHT	Hilbert Huang transform
HS	Hilbert spectrum
IF	instantaneous frequency
IMF	intrinsic mode function
MF	marginal frequency
MI	motor imagery
PCC	Pearson’s correlation coefficient
RF	residue functions
RTE	refereed total energy
TF	time-frequency
